# Helping patients find their way to better diabetic retinopathy care

**Published:** 2012

**Authors:** Silvana Faillace, P Kotha Satya, Widya Prasetyanti, Ikke Sumantri, Gitalisa Andayani Adriono, Gitalisa Ari Djatikusumo Adriono, Anggun Rama Yudantha, Imam Subekti, Em Yunir, Budiman

**Affiliations:** Helen Keller International, Indonesia; Department of Ophthalmology, RSCM Hospital, Jakarta, Indonesia; Department of Internal Medicine, RSCM Hospital, Jakarta, Indonesia

In order to effectively address diabetic retinopathy (DR), people with diabetes must understand that diabetes can damage their eyes. They must know where to go for tests to determine whether they have DR. If they have DR, they must be told, and referred to someone who is able to provide treatment at an affordable cost. Those who do not have DR must be informed that they need annual follow-up and be reminded to return at the appropriate time.

This can be very difficult to achieve in practice, as patients with diabetes are usually treated in a primary health care setting or in specialist clinics, such as endocrinology clinics. The medical staff providing diabetes care may not have the knowledge or skills to inform patients about DR or to do any screening.

Although the prevalence of DR has not been established in Indonesia, the country currently has over 9 million people with diabetes. At Cipto Mangunkusomo Hospital, a national referral hospital in Indonesia, the department of ophthalmology and the department of internal medicine are working together to create an integrated DR programme.

When diabetes patients attend the endocrinology clinic (part of the department of internal medicine) they are given information on DR and offered screening tests (visual acuity and retinal photography). These tests now form an integral part of the examination protocol for all diabetes patients visiting the endocrinology clinic. New patients are offered the tests upon diagnosis, and existing patients are tested once a year. In addition, all diabetes patients are given a checklist with all the tests they need, and visual acuity and retinal photography are included on this list.

**Figure:**
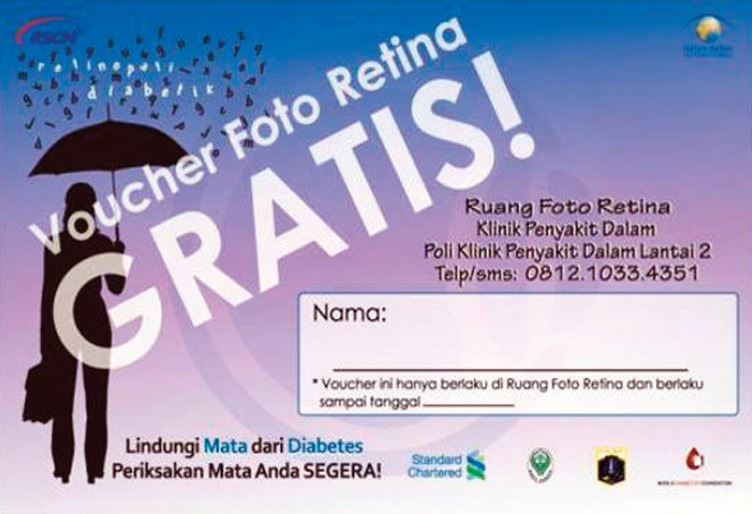
Health workers have started to distribute screening vouchers to people with diabetes in the community. The vouchers encourage people to come to Cipto Mangunkusomo hospital for free diabetic retinopathy screening tests.

**Figure:**
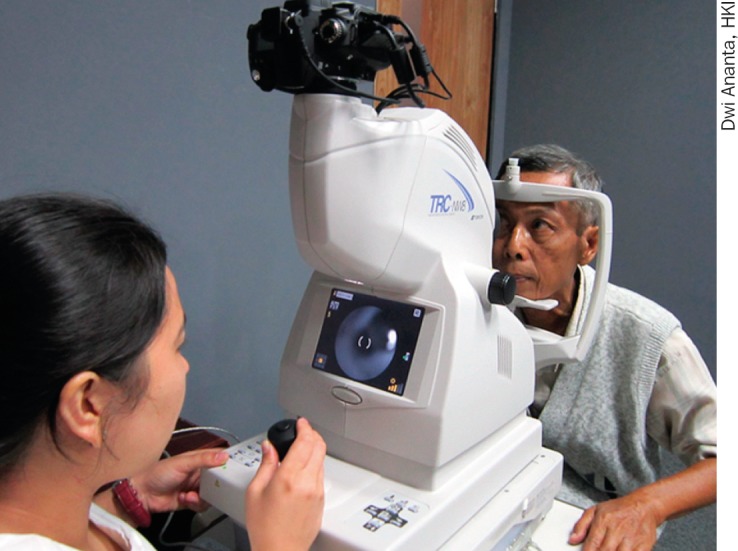
Screening and photo grading services are provided free of charge. INDONESIA

Screening and retinal photo grading services are provided free. The cost of laser treatment depends on the patient's insurance scheme, and is free for those without health insurance.

Keeping track of patients who have been identified with diabetic retinopathy is challenging, particularly as this involves the two different departments: internal medicine and ophthalmology. As a result, a DR programme registration card was introduced. This card is given to all the patients who are screened at the internal medicine clinic. If referred to ophthalmology, the patient brings this card to the eye clinic. The ophthalmology patient number is also indicated on the card. This helps identify patients that have been screened by the DR programme and makes it possible to track patients if they need to be followed up at the eye clinic.

In July 2011, the eye clinic was moved to a new location, approximately one kilometre away from the screening room. It was anticipated that it would be difficult for patients to find the new location. Sign boards directed patients from the internal medicine department to the new eye clinic. One of the members of the team also accompanied the patients to the new centre.

Since November 2011, all patients with DR are telephoned immediately after grading, irrespective of their priority level. The high priority patients are followed up more intensely: if the patients do not return for the appointment, they are contacted again within three days of their appointment. The programme also gives the endocrinology clinic a list of patients with sight-threatening DR who have not returned for treatment. The clinic then ensures that these patients receive further education during their regular clinic visits and are encouraged to return for laser treatment. This has increased the number of returning patients.

From July 2010 to December 2011, 3,762 diabetic patients have been screened and 861 identified with DR. Of the total number of patients screened, 1,462 had other eye conditions. The programme has also trained 67 community-based diabetes educators and 190 community health workers focusing on diabetes and eye disease. Thirty five ophthalmologists have been trained in laser surgery. Over four hundred internists, ophthalmology residents, and clinic staff have been educated about the importance of DR. The programme was also supported by radio and newspaper campaigns high lighting the importance of eye health and diabetes.

It has taken a lot of effort to ensure the two departments are able to work together successfully. The plan for an integrated DR service was first discussed with senior clinic administrators and a plan of action was then agreed upon and followed up. Multiple educational sessions were held for staff, nurses, clerks, and internists at the clinic. Even so, constant communication and awareness-building with administrative and medical departments is still necessary.

The programme receives technical assistance and external funding from Helen Keller International.

